# Imperatorin Alleviates Intestinal Fibrosis by Suppressing AIM2-mediated GSDMD Pyroptosis in Macrophages

**DOI:** 10.1016/j.jcmgh.2025.101625

**Published:** 2025-09-05

**Authors:** Sheng Li, Fangqing Zhu, Yao Xie, Teng Ben, Ke Liu, Xinlong Lin, Qian Zhou, Yin Zhang, Xinyue Zhang, Yeling Chen, Yuexin Ren, Xianfei Wang, Fachao Zhi, Gao Tan

**Affiliations:** 1 Guangdong Provincial Key Laboratory of Gastroenterology, Institute of Gastroenterology of Guangdong Province, Department of Gastroenterology, Nanfang Hospital, Southern Medical University, Guangzhou, China; 2 Department of Gastroenterology, Yuebei People’s Hospital, Shantou University Medical College, Shaoguan, China; 3 Department of Gastroenterology, Ganzhou Hospital Affiliated to Nanfang Hospital, Southern Medical University, Ganzhou, China; 4 Department of Gastroenterology, the Second Affiliated Hospital, University of South China, Hengyang, China; 5 Department of Gastroenterology, Digestive Endoscopy Center, Affiliated Hospital of North Sichuan Medical College, Nanchong, China

**Keywords:** Crohn’s disease, Imperatorin, Intestinal fibrosis, Pyroptosis

## Abstract

**Background & Aims:**

Over-activation of pyroptosis, recently reidentified as Gasdermin D (GSDMD)-mediated proinflammatory cell death, results in severe inflammation-related disorders. Intestinal fibrosis, an inflammation-related disorder, remains one of the most common and intractable complications of Crohn’s disease (CD). However, it is unknown whether excessive pyroptosis contributes to the development of intestinal fibrosis in CD.

**Methods:**

Immunofluorescence costaining of CD11b and the pyroptosis-inducing fragment GSDMD-N terminal (GSDMD-NT) was performed in stenotic ileocecal valve tissues from patients with CD. A 2,4,6-trinitrobenzenesulfonic acid (TNBS)-induced mouse CD model was established. J744a.1 macrophages pretreated with imperatorin (IMP) were transfected with lipopolysaccharides (LPS) plus poly (dA: dT) to explore potential regulatory mechanisms controlling GSDMD-mediated pyroptosis in vitro.

**Results:**

GSDMD-NT^+^ CD11b^+^ macrophages were significantly increased in stenotic ileocecal valve tissues from patients with CD compared with that in normal ileocecal valve tissues, reflecting that GSDMD-mediated pyroptosis in macrophages is excessively activated in CD-associated intestinal fibrosis. In the TNBS-induced model, *Gsdmd*^-/-^ mice had decreased intestinal fibrosis compared with their wild-type littermates. We also found that imperatorin (IMP), a natural furocoumarin, not only alleviated TNBS-induced intestinal fibrosis, but also inhibited TNBS-induced increase of AIM2 expression, Caspase-1 activation, and GSDMD cleavage in the colon. In vitro, we revealed IMP acting as a new regulatory factor that negatively controlled the AIM2 inflammasome via downregulating AIM2 expression, thereby avoiding excessive GSDMD-mediated pyroptosis in J744a.1 macrophages.

**Conclusions:**

IMP negatively controls GSDMD-mediated pyroptosis via inhibiting the AIM2 pathway in macrophages. Thus, IMP enema may be a potential therapeutic approach for CD-associated intestinal fibrosis.

## Graphical abstract

**Figure:**
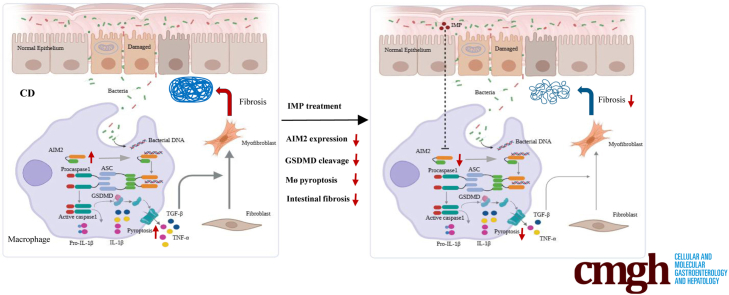

**Summary**

We discovered imperatorin (IMP), a natural compound, alleviates Crohn’s intestinal fibrosis by blocking AIM2-mediated pyroptosis in macrophages. Validated in human tissues and animal models, IMP enema offers a novel targeted therapy for stricturing complications.

Crohn’s disease (CD) is a kind of refractory chronic inflammatory bowel diseases (IBD) of unknown etiology.^1^ With increasing incidence worldwide, it has become a global public health problem.^2^ In the early stages, its main manifestation is intestinal inflammation, whereas in the middle and late stages, with repeated episodes of intestinal inflammation, it gradually develops into intestinal strictures.^3^ After 10-year diagnosis of CD, approximately 70% of patients will develop intestinal strictures, most of which require endoscopic balloon dilation and surgery, despite a high recurrence rate.^4^ The overall prognosis of patients with CD with intestinal strictures is extremely poor.^5^ The essence of intestinal strictures is intestinal fibrosis, but specific antifibrotic drugs are currently unavailable.^3^ Although intestinal inflammation in patients with CD has been somewhat controlled in clinical treatment with the rise of immunosuppressants and biologics, these drugs fail to halt fibrotic progression.^6^ Thus, elucidating fibrogenic mechanisms and identifying novel therapeutics are urgent clinical priorities.

Intestinal fibrosis is characterized by excessive deposition of the extracellular matrix (ECM) in the intestine.^3^^,^^6^ To date, known pathological processes responsible for the development of intestinal fibrosis include fibroblast proliferation, migration, recruitment and activation, fibrocyte recruitment, activation and differentiation of stellate cells, pericyte differentiation, EMT, and EndoMT.^7^ Recurrent inflammation is regarded to be the main cause of intestinal fibrosis in CD, because it participates in these pathological processes from multiple aspects.^7^, ^8^, ^9^ Certain inflammation and consequent fibroblast activation are necessary for normal physiological repair of damaged tissues, whereas recurrent and excessive inflammation will initiate multiple pathological processes that result in dysregulated repair and tissue fibrosis.^10^ However, what mechanism induces excessive inflammation contributing to the development of intestinal fibrosis is unclear.

Pyroptosis is initially regarded as Caspase1-dependent cell death in response to certain microbial invasion.^11^ With the recent identification of Gasdermin D-N terminal (GSDMD-NT) as the pyroptotic executioner, pyroptosis is redefined as Gasdermin-mediated pro-inflammatory programmed cell death, characterized by cell membrane swelling and rupture, and distinct from apoptosis, autophagy, ferroptosis, cuproptosis, and necroptosis in morphological characteristics.^12^, ^13^, ^14^, ^15^, ^16^, ^17^ Inflammasomes, including NLRP1, NLRP3, NLRC4, AIM2, and Pyrin, play a vital role in triggering pyroptosis.^16^ Among them, the AIM2 inflammasome in macrophages sensing microbial DNA activates Caspase1 and subsequently induces Gasdermin D (GSDMD)-mediated pyroptosis, thereby acting as an anti-microbial host defense, but strict negative regulatory mechanisms are required to avoid excessive activation of AIM2-dependent and GSDMD-mediated pyroptosis, which is harmful to the host.^18^ Currently, it is a well-known fact that patients with CD have an exaggerated intestinal inflammatory response to invaded microbes.^19^ However, it is unclear whether it is due to impaired negative regulatory mechanisms controlling the AIM2 inflammasome that invading microbes trigger excessive GSDMD-mediated pyroptosis in macrophages, leading to severe intestinal inflammation and fibrosis in CD.

Natural compounds represent promising anti-fibrotic candidates; among them, imperatorin (IMP)—a bioactive furanocoumarin—has been shown to attenuate fibrosis in extra-intestinal organs^20^ and modulate inflammatory caspases,^21^ yet its therapeutic potential for intestinal fibrosis remains unexplored. This study aimed to: (1) determine whether AIM2-GSDMD pyroptosis promotes intestinal fibrosis in CD; and (2) evaluate IMP’s efficacy and mechanism of action targeting the AIM2 inflammasome. Through integrated analysis of clinical specimens, murine models, and in vitro systems, we demonstrate that IMP alleviates fibrosis by suppressing AIM2-mediated pyroptosis in macrophages, thereby proposing a novel therapeutic strategy for CD-associated intestinal fibrosis.

## Results

### GSDMD-mediated Pyroptosis in Macrophages Is Excessively Activated in Intestinal Stenotic Tissues From Patients With CD

**Figure 1:**
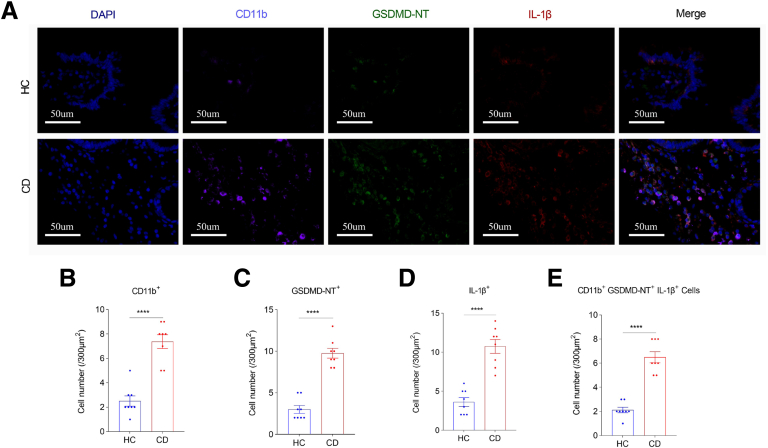
**CD11b^+^ GSDMD-NT^+^ IL-1β^+^ macrophages are increased in stenotic ileocecal valve tissues from patients with CD.** (*A–E*) Triple immunofluorescence costaining of CD11b, GSDMD-NT, and IL-1β in stenotic ileocecal valve tissues from patients with CD and in normal ileocecal valve tissues from HCs. (*A*) Representative immunofluorescence images. Scale bars:50 μm. (*B–E*) The number of CD11b^+^, GSDMD-NT^+^, and IL-1β^+^ cells per 300 μm^2^. Pyroptotic macrophages were costaining with CD11b (*pink*), GSDMD-NT (*green*), and IL-1β (*red*). CD11b^+^ GSDMD-NT^+^ IL-1β^+^ macrophages were counted under a microscope. Data represent means ± SEM (n = 8 per group); ∗∗∗∗*P* < .0001 by Student’s *t*-test.

To investigate whether GSDMD-mediated pyroptosis in macrophages is associated with CD-associated intestinal fibrosis, we performed triple immunofluorescence staining of CD11b, GSDMD-NT, and interleukin (IL)-1β in stenotic ileocecal valve tissues from patients with CD and in normal ileocecal valve tissues from healthy controls (HCs), and found that CD11b^+^ cells, GSDMD-NT^+^ cells, IL-1β^+^ cells, and CD11b^+^ GSDMD-NT^+^ IL-1β^+^ cells were significantly increased in stenotic ileocecal valve tissues from patients with CD compared with that in normal ileocecal valve tissues from HCs, suggesting that GSDMD-mediated pyroptosis in CD11b^+^ macrophages of intestinal stricture tissues is excessively activated in CD (Figure 1*A–E*).

**Figure 2:**
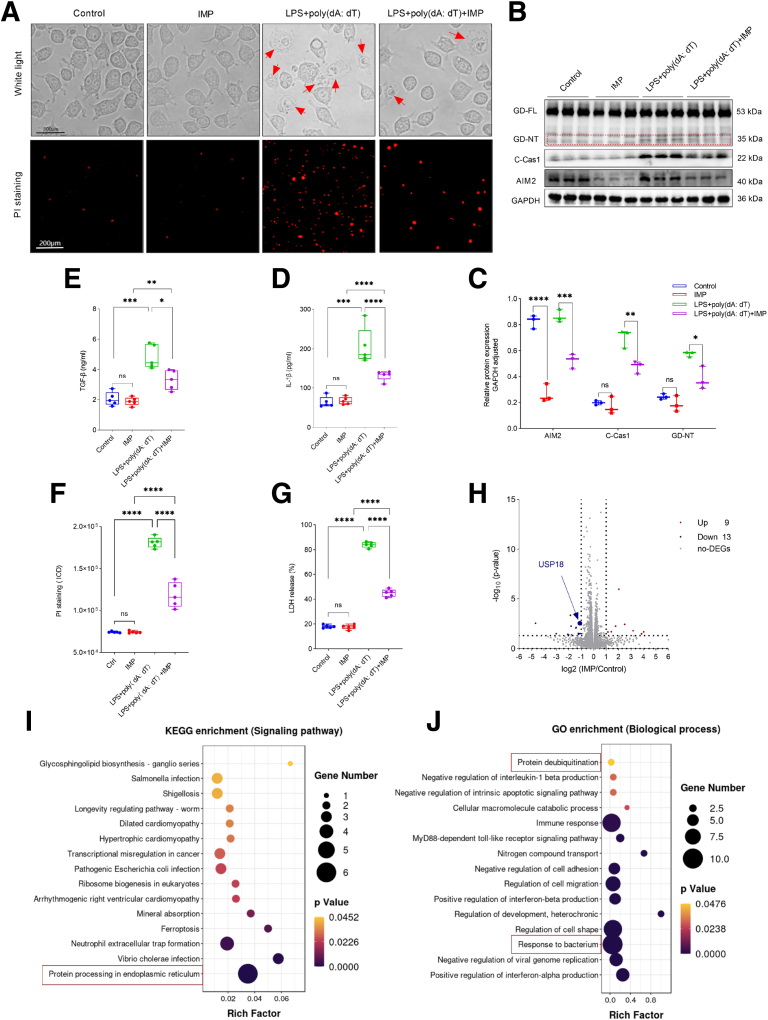
**IMP inhibits GSDMD-mediated pyroptosis in macrophages through downregulating AIM2 expression.** (*A–G*) J774A.1 macrophages were treated with or without 50 μM IMP for 6 hours following transfection with 1 μg/μL LPS plus 2.5 ug/mL poly (dA: dT) for 8 hours. Control represents untreated cells. (*B, C*) Their whole cell lysates were subjected to Western blot (WB) analysis. (*B*) Representative WB images. Scale bars: 100 μm. *Red arrows*: pyroptotic bubbles. (*C*) The numbers present the intensity ratio of indicated proteins/GAPDH analyzed by ImageJ software. (*D, G*) Their culture supernatants were collected for LDH and IL-1β assays. (*E*) Their culture supernatants were collected to stimulate the human colon fibroblast cell line CCD-18Co. After 24 hours, CCD-18Co cells were changed to a new medium and cultured for 12 hours, and subsequently their culture supernatants were collected for TGF-β assays. (*F*) These cells were stained with PI solution (100 μL/well) at room temperature for 15 minutes in the dark, and then observed under a fluorescence microscope. Quantitative analysis of PI staining by ImageJ software. IOD, integrated optical density. (*H–J*) J744a.1 macrophages were treated with 50 μM IMP for 6 hours, then total RNA was isolated and DEGs were analyzed by RNA-seq. Control represents untreated cells. (*H*) Volcano plot showing the DEGs in the IMP-treated group vs the untreated control group. Genes were plotted based on log_2_ fold change against the absolute confidence (−log_10_*p* value). *Green and red dots* represent down- and up-regulated genes, respectively. (*I*) KEGG pathway enrichment analysis of the DEGs in the 2 groups. Dot size represents the gene number of DEGs and the corresponding color represents its *P* value. (*J*) GO functional enrichment analysis of the DEGs in the 2 groups. Enriched GO terms according to biological processes. (*C–G*) Data represent means ± SD from 3 to 5 technical replicates; ns, not significant; ∗*P* < .05, ∗∗*P* < .01, ∗∗∗*P* < .001, ∗∗∗∗*P* < .0001 by 1-way ANOVA test. All data shown are representative of 3 independent experiments.

To further determine whether GSDMD-mediated pyroptosis in macrophages participates in the development of intestinal fibrosis, we first stimulated J774A.1 macrophages with lipopolysaccharide (LPS) plus poly (dA: dT) and then used their culture supernatants to stimulate CCD-18Co fibroblasts in vitro. We observed that J774A.1 cells treated with LPS plus poly (dA: dT) had obvious pyroptotic bubbles, Caspase-1 activation, and GSDMD cleavage, and in their culture supernatants, the concentration of IL-1β was significantly increased (Figure 2*A–D*), suggesting that LPS plus poly (dA: dT) can induce GSDMD-mediated pyroptosis in macrophages to secrete lots of cytokines, including IL-1β. In addition, we also found that the concentration of transforming growth factor beta (TGF-β) was significantly increased in the culture supernatants from CCD-18Co cells treated with the supernatants of pyroptotic macrophages compared with that from untreated CCD-18Co cells (Figure 2*E*). These results suggest that GSDMD-mediated pyroptosis in macrophages secrete lots of cytokines, such as IL-1β, to activate intestinal fibroblasts, thereby promoting the occurrence and development of intestinal fibrosis.

### Gsdmd Deletion Alleviates 2,4,6-trinitrobenzenesulfonic Acid-induced Colitis and Fibrosis in Mice

**Figure 3:**
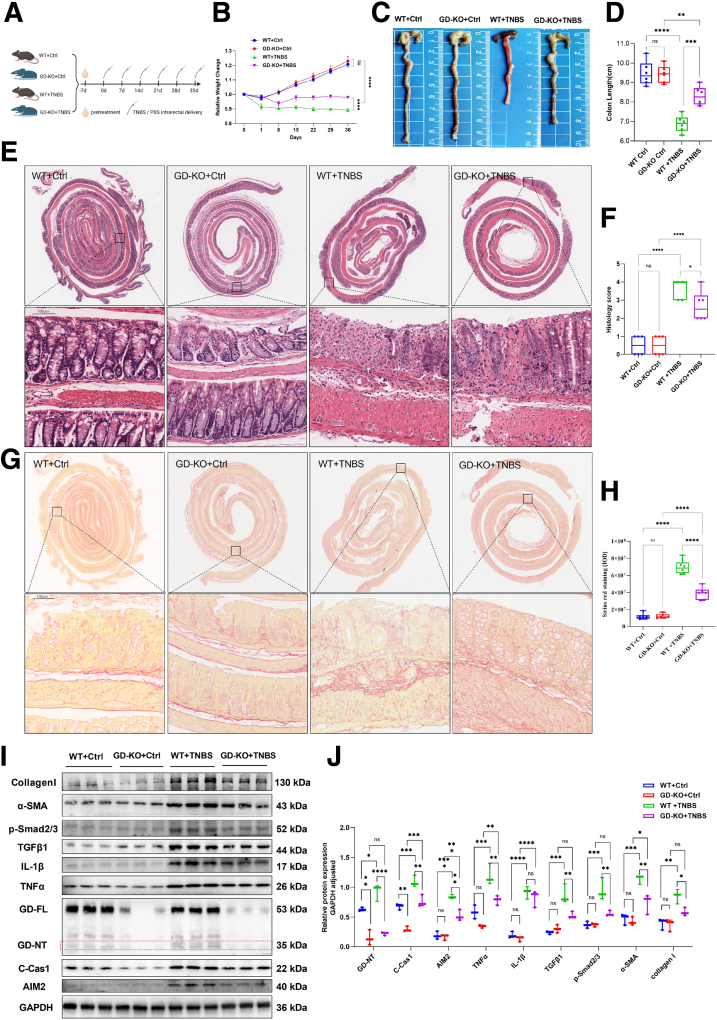
***Gsdmd*^-/-^ mice are less sensitive to TNBS-induced colitis and fibrosis.** (*A–J*) *Gsdmd*^-/-^ (KO) mice and WT littermates were intrarectally injected with 2.5% TNBS to induce experimental chronic CD model, or with PBS enema as a negative control (Ctrl). On the 36th dday, all the mice were euthanized, and specimens were taken for testing. (*B*) One day after TNBS or PBS enema, body weight changes were monitored. (*C, D*) Representative images of the colons. Colon lengths were measured. (*E, F*) Colon sections were examined histologically. (*E*) Representative images of the H&E-stained colon sections; *black boxes* represent enlarged images. Scale bars: 100 μm. (*F*) Histology scores for colonic inflammation were measured. (*G, H*) Representative images of Sirius red staining; *black boxes* represent enlarged images. Scale bars: 100 μm. Quantitative analysis by ImageJ software. (*I, J*) The levels of indicated proteins in the colonic tissues were analyzed by Western blot (WB). (*I*) Representative WB images. (*J*) The numbers present the intensity ratio of indicated proteins/GAPDH analyzed by ImageJ software. (*B, D, F, H*) Data represent means ± SEM (n = 6 per group); ns, not significant; ∗*P* < .05, ∗∗*P* < .01, ∗∗∗*P* < .001, ∗∗∗∗*P* < .0001 by 1-way ANOVA test. All data shown are representative of 3 independent experiments.

To determine the role of GSDMD-mediated pyroptosis in CD-associated fibrosis, *Gsdmd*^*-/-*^ mice and their wild-type (WT) littermates were rectally injected with 2,4,6-trinitrobenzenesulfonic acid (TNBS) to induce the chronic CD model according to the methods shown in Figure 3*A*. Compared with WT littermates, *Gsdmd*^*-/-*^ mice were resistant to TNBS-induced colitis and intestinal fibrosis, exhibiting less weight loss, colonic shortening, inflammation-associated histological scores, Sirius red staining, and decreased expression of Collagen I, alpha smooth muscle actin (α-SMA), p-Smad2/3, TGFβ1, and tumor necrosis factor alpha (TNFα) in the TNBS colons (Figure 3*B-J*). In addition, we found that *Gsdmd* knockout significantly decreased the levels of AIM2 expression, cleaved-Caspase1, and GSDMD-NT in the TNBS colons (Figure 3*I, J*). Together, these results suggest that Gsdmd-mediated pyroptosis plays an important role in the development of TNBS-induced colitis and intestinal fibrosis.

### IMP Alleviates TNBS-induced Colitis and Fibrosis Through Inhibiting GSDMD-mediated Pyroptosis

**Figure 4:**
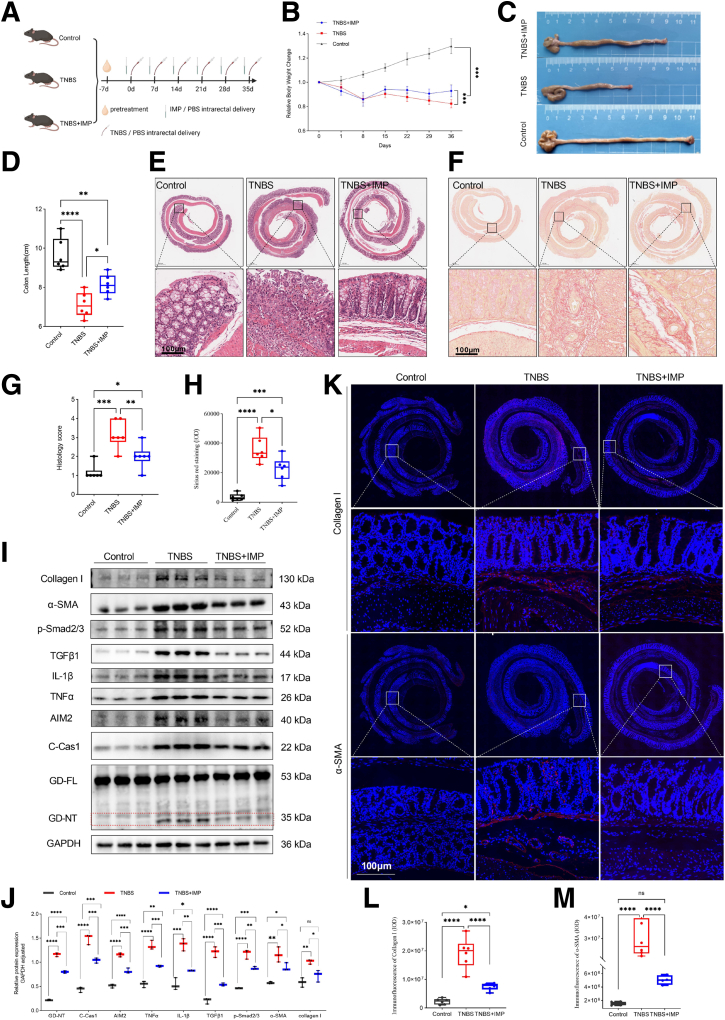
**IMP alleviates TNBS-induced colitis and fibrosis in mice.** (*A–M*) Littermate WT mice were divided into 3 groups. In the Control group, the mice were intrarectally injected with PBS alone. One day before TNBS enema, the mice in the TNBS group were intrarectally injected with 100 μL PBS, whereas the mice in the TNBS + IMP group were injected with 100 μL 3 mM IMP. On the 36th day, all mice were euthanized, and specimens were taken for testing. (*B*) One day after TNBS or PBS enema, body weight changes were monitored. (*C, D*) Representative images of the colons. Colon lengths were measured. (*E, G*) Colon sections were examined histologically. (*E*) Representative images of the H&E-stained colon sections; *black boxes* represent enlarged images. Scale bars: 100 μm. (*G*) Histology scores for colonic inflammation were measured. (*F, H*) Representative images of Sirius red staining; *black boxes* represent enlarged images. Scale bars: 100 μm. Quantitative analysis by ImageJ software. (*I, J*) The levels of indicated proteins in the colonic tissues were analyzed by Western blot (WB). (*I*) Representative WB images. (*J*) The numbers present the intensity ratio of indicated proteins/GAPDH analyzed by ImageJ software. (*K–M*) Representative immunofluorescence images of Collagen Ⅰ and α-SMA immunostaining in the colonic tissues; *white boxes* represent enlarged images. Scale bars: 100 μm. Quantitative analysis by ImageJ software. (*B, D, G, H, L, M*) Data represent means ± SEM (n = 6 per group); ns, not significant; ∗*P* < .05, ∗∗*P* < .01, ∗∗∗*P* < .001, ∗∗∗∗*P* < .0001 by 1-way ANOVA test. All data shown are representative of 3 independent experiments.

Traditional Chinese medicine (TCM) is a great treasure house of medicine, in which searching for natural compounds that may suppress excessive pyroptosis has become a potent and valuable approach for inflammation-related disorders, such as fibrosis and cancer. A recent finding shows that IMP, a natural furocoumarin, can alleviate pulmonary fibrosis induced by bleomycin in vivo.^20^ Another study displays that it can inhibit obesity-induced excessive activation of Caspase-1 in stellate sympathetic ganglion,^22^ whereas activated Caspase-1 can cleave GSDMD into C- and N-terminal, in which GSDMD-NT, as the pyroptotic executioner, induces proinflammatory cell death.^16^ Thus, we speculated that IMP may alleviate CD colitis and fibrosis via inhibiting excessive activation of Caspase-1-dependent and GSDMD-mediated pyroptosis. To test it, we rectally injected IMP or phosphate buffered saline (PBS) as a control in the TNBS-induced chronic CD model according to the methods shown in Figure 4*A*. The IMP-administered WT mice were weakly responsive to TNBS-induced colitis and intestinal fibrosis compared with the controls, displaying reduced weight loss, colonic shortening, inflammation-associated histological scores, Sirius red staining, and downregulated colonic expression of Collagen I, α-SMA, p-Smad2/3, TGFβ1, IL-1β, and TNFα, as well as decreased immunofluorescence staining of Collagen I and α-SMA in the colons (Figure 4*B–M*). These results suggest that IMP plays a protective role against CD-associated inflammation and fibrosis.

Our previous finding showed that disruption of the intestinal epithelial cell (IEC) barrier causes an increase of intestinal mucosal bacteria, thus activating abnormal inflammatory responses in the gut,^21^ in line with a generally accepted view that an excessive intestinal immune response to the gut microbe in CD is due to disruption of the IEC barrier.^19^ The AIM2 inflammasome sensing bacterial DNA triggers GSDMD-mediated pyroptosis in macrophages against bacterial invasion, but a strict regulatory mechanism is required to avoid its excessive activation by bacterial DNA, which is harmful to the host.^18^ Here, we found that the TNBS-challenged mice had obvious epithelium damage and crypt loss, and increased AIM2 expression in the colon (Figure 4*E, I, J*), suggesting that TNBS caused excessive AIM2 activation upon various intestinal luminal bacteria translocation into the mucosa from disrupted IEC barrier. In addition, TNBS also caused obvious Caspase1 activation and Gsdmd cleavage to produce the pyroptosis-inducing fragment GSDMD-NT (Figure 4*I, J*). However, IMP significantly decreased the levels of AIM2 expression, cleaved Caspase1, and GSDMD-NT in the TNBS colons (Figure 4*I, J*). Together, these results indicate that IMP acts as a negative regulatory factor controlling excessive AIM2 and Caspase1 activation and subsequent GSDMD-mediated pyroptosis.

### IMP Negatively Controls GSDMD-mediated Pyroptosis in Macrophages Through Inhibiting the AIM2 Pathway

To confirm the role of IMP in GSDMD-mediated pyroptosis, J774A.1 macrophages were pretreated with or without IMP, then transfected with LPS plus poly (dA: dT) in vitro. We observed that the cells transfected with LPS plus poly (dA: dT) had obvious pyroptotic bubbles with disrupted cytomembranes as reflected by increased lactate dehydrogenase (LDH) release and propidium iodide (PI) staining compared with the untransfected cells (Figure 2*A, F, G*). Moreover, this treatment also caused Caspase1 activation and GSDMD cleavage (Figure 2*B, C*), indicating that it induces GSDMD-mediated pyroptosis. However, IMP inhibited GSDMD-mediated pyroptosis induced by LPS plus poly (dA: dT), displaying less pyroptotic bubbles, LDH release, PI staining, AIM2 expression, cleaved-Caspase1, and GSDMD-NT in the cells with IMP pretreatment compared with the cells without IMP pretreatment (Figure 2*A–C, F, G*). Furthermore, IMP alone downregulated AIM2 expression, but not GSDMD or Caspase1 (Figure 2*B, C*). These results suggest that IMP inhibits GSDMD-mediated pyroptosis through downregulating AIM2 expression in macrophages.

**Figure 5:**
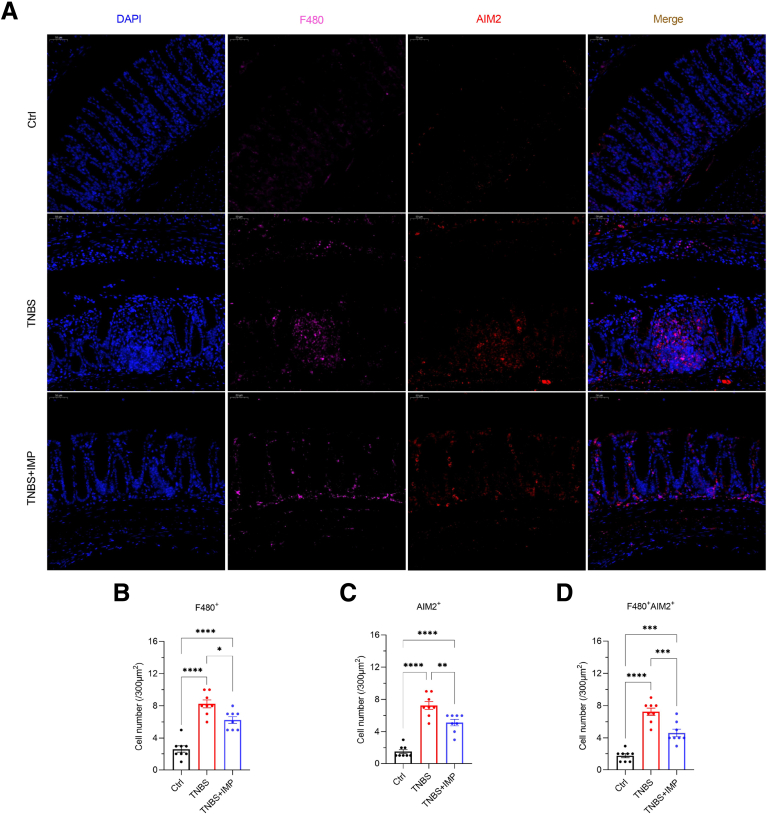
**IMP alleviates GSDMD-mediated pyroptosis through downregulating AIM2 expression in colonic macrophages.** (*A–D*) Double immunofluorescence staining of F4/80 and AIM2 in colon tissues from TNBS-induced colitis mice with/without IMP intervention and control groups. (*A*) Representative confocal micrographs showing costaining patterns. Scale bar: 50 μm. (*B–D*) Quantitative analysis of immunopositive cells within 300 μm^2^ regions. (*B–E*) Data represent means ± SEM (n = 10 per group); ∗*P* < .05, ∗∗*P* < .01, *∗∗∗P* < .001, ∗∗∗∗*P* < .0001 by 1-way ANOVA test.

To further validate the mechanism by which IMP alleviates GSDMD-mediated macrophage pyroptosis via AIM2 in mice, we performed double immunofluorescence staining for F4/80 and AIM2 on colon tissues from IMP-treated TNBS-induced colitis (CD) mice. The results demonstrated that, compared with the control group, the TNBS group exhibited a significant increase in F4/80^+^ macrophage density along with elevated AIM2^+^ cell density. Double colocalization analysis revealed a marked increase in F4/80^+^ AIM2^+^ double-positive cells in the TNBS group relative to controls (Figure 5*A–D*). In contrast, the TNBS + IMP cotreatment group showed reduced F4/80^+^ macrophage density, decreased AIM2^+^ cell density, and fewer F4/80^+^ AIM2^+^ double-positive cells compared with the TNBS group, indicating that IMP mitigates GSDMD-mediated pyroptosis in vivo by downregulating AIM2 expression in macrophages. Taken together, these results suggest that the AIM2 inflammasome emerges as the pivotal protein through which IMP regulates macrophage pyroptosis.

### AIM2 Upregulation in Macrophages Associates With Intestinal Fibrosis in CD

**Figure 6:**
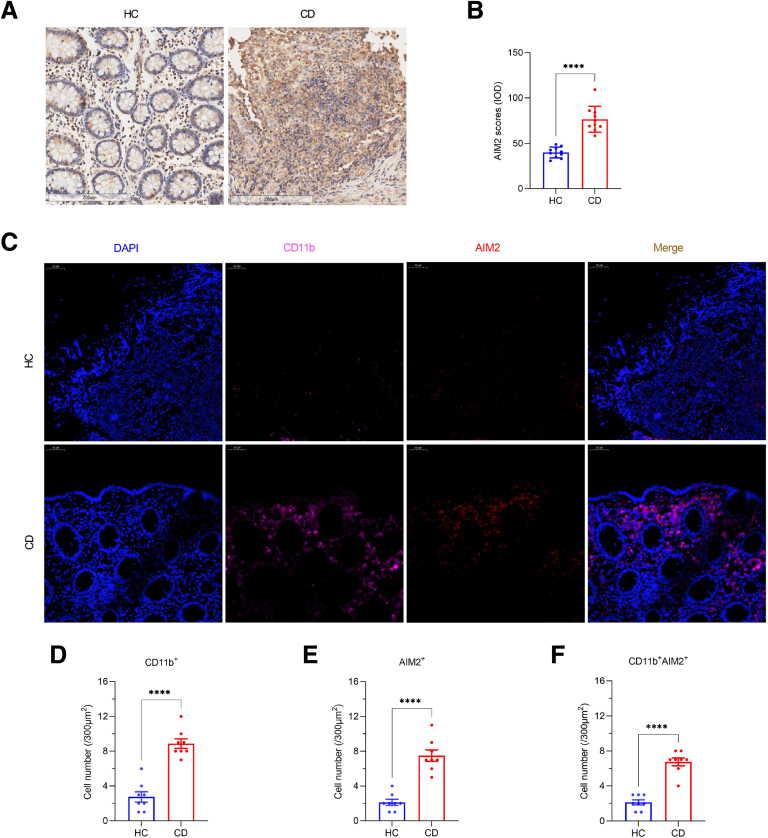
**AIM2 expression is upregulated in stenotic ileocecal valve tissues from patients with CD.** (*A–B*) IHC analysis of AIM2 levels in stenotic ileocecal valve tissues from patients with CD and in normal ileocecal valve tissues from HCs. (*A*) Representative IHC images. Scale bars: 200 μm. (*B*) Quantitative analysis by Image-Pro Plus software. IOD, integrated optical density. (*C–F*) Double immunofluorescence costaining of CD11b and AIM2 in stenotic ileocecal valve tissues from patients with CD and in normal ileocecal valve tissues from HCs. (*C*) Representative immunofluorescence images. Scale bars: 50 μm. (*D–F*) The number of CD11b^+^ and AIM2^+^ cells per 300 μm^2^. CD11b^+^ AIM2^+^ macrophages were counted under a microscope. (*B, D–F*) Data represent means ± SEM (n = 8–10 per group); ∗∗∗∗*P* < .0001 by Student’s *t*-test.

To further elucidate the role of AIM2 in the pathogenesis of CD-associated intestinal fibrosis, we performed immunohistochemical (IHC) and immunofluorescence staining of AIM2 in stenotic ileocecal valve tissues from patients with CD and in normal ileocecal valve tissues from HCs. We found that AIM2 expression in stenotic ileocecal valve tissues from patients with CD is significantly increased compared with that in normal ileocecal valve tissues from HCs (Figure 6*A–B*). In addition, we found significantly increased density of CD11b^+^ macrophages and elevated AIM2^+^ cell density in CD specimens compared with the HC group. Dual colocalization analysis revealed a marked increase in CD11b^+^ AIM2^+^ double-positive cells in patients with CD vs HCs (Figure 6*C–F*). Integrated with prior findings (Figure 1), these results indicate that pathogenic stimuli trigger macrophage AIM2 overexpression in humans, which subsequently drives excessive GSDMD-mediated pyroptosis, ultimately exacerbating intestinal fibrosis and stenosis in CD.

## Discussion

This study elucidates a novel mechanism by which IMP alleviates intestinal fibrosis in CD through suppression of AIM2-mediated pyroptosis in macrophages. We provide multi-level evidence demonstrating that excessive GSDMD-dependent pyroptosis drives fibrotic progression in CD and establish AIM2 inflammasome inhibition as the primary therapeutic mechanism of IMP. Critically, clinical specimens from patients with CD with ileocecal stenosis revealed significant enrichment of CD11b^+^ GSDMD-NT^+^ macrophages (Figure 1), implicating pyroptotic hyperactivity in human disease pathogenesis. This observation was functionally validated in vivo, where genetic ablation of Gsdmd markedly attenuated intestinal fibrosis in a TNBS-induced chronic colitis model (Figure 3). The anti-fibrotic efficacy of IMP was consistently demonstrated through its ability to restore colon architecture, downregulate fibrotic markers (Collagen I, α-SMA), and suppress inflammatory cytokines (IL-1β, TNF-α) (Figure 4). Crucially, IMP administration significantly reduced AIM2 protein expression, Caspase-1 activation, and GSDMD cleavage in colonic tissues (Figure 4*I–J*), whereas in vitro studies confirmed direct inhibition of AIM2-mediated pyroptosis in macrophages (Figure 2).

The centrality of AIM2 as a key regulatory node is further underscored by spatial analyses. In TNBS-challenged mice, IMP treatment reduced F4/80^+^ AIM2^+^ macrophage infiltration (Figure 5), paralleling reduced pyroptosis and fibrosis. Importantly, human stenotic tissues exhibited elevated CD11b^+^ AIM2^+^ macrophages compared with HCs (Figure 6), establishing clinical relevance for this pathway. To explore the potential mechanism underlying AIM2 downregulation by IMP in macrophages, RNA sequencing of J744a.1 cells with or without IMP stimulation was performed to uncover differentially expressed genes (DEGs). There were 9 upregulated DEGs in the IMP group compared with the control group, and 13 downregulated DEGs (Figure 2*H*). Kyoto Encyclopedia of Genes and Genomes (KEGG) analysis revealed the functional enrichment of protein processing in endoplasmic reticulum, a post-transcriptional modification (Figure 2*I*). Moreover, Gene Ontology (GO) analysis revealed the functional enrichment of protein deubiquitination (Figure 2*J*). Among the DEGs, the *USP18* protein is a deubiquitinating enzyme (Figure 2*H*). Although these results suggest that IMP may regulate AIM2 protein stability through the deubiquitinating enzyme pathway, such as an USP18-dependent post-transcriptional manner, the precise molecular mechanism warrants further investigation.

Our results reshape understanding of macrophage-driven fibrogenesis. Conventionally, fibrosis is attributed to M2-polarized macrophages via TGF-β1 secretion.^23^ However, we demonstrate that pyroptosis-associated cytokine release (eg, IL-1β) directly activates fibroblasts to produce TGF-β (Figure 2*E*),^24^ creating a feed-forward loop independent of polarization states.^25^ This aligns with observations that pyroptotic macrophages release copious IL-1β and TNF-α,^21^ which are known fibroblast mitogens and inducers of endothelial-mesenchymal transition.^26^ Thus, dysregulated pyroptosis intensity, rather than macrophage polarization per se, emerges as a critical determinant of fibrotic outcomes.^27^ IMP’s ability to fine-tune this axis (reducing pathologic pyroptosis while preserving microbial defense) offers a strategic advantage over global immunosuppressants.

Several limitations merit consideration. First, the absence of macrophage-specific AIM2 knockout data limits mechanistic depth, though IMP’s efficacy across models supports target engagement.^28^ Second, although we established AIM2 as IMP’s functional target, its direct molecular binder (whether AIM2 itself or upstream regulators) remains undefined.^29^ Third, cell-type-specific contributions (eg, IECs or fibroblasts) to IMP’s anti-fibrotic effects require elucidation using conditional knockout models. Four, the translational potential of IMP enema necessitates further pharmacokinetic and safety studies. Nevertheless, our work provides foundational insights: (1) GSDMD-mediated pyroptosis is mechanistically linked to CD-associated intestinal fibrosis; (2) AIM2 inflammasome activation is a pathogenic trigger; and (3) IMP represents a promising therapeutic agent acting through AIM2 suppression.

7In conclusion, we identify hyperactivated AIM2-GSDMD pyroptosis as a key driver of intestinal fibrogenesis in CD. By demonstrating IMP’s efficacy in targeting this pathway—evidenced by reduced AIM2 expression, diminished pyroptosis, and attenuated fibrosis across human, murine, and cellular models—our study nominates AIM2 inhibition as a viable strategy for treating CD-associated intestinal fibrosis. Future efforts should focus on delineating the molecular interface of IMP-AIM2 interaction and advancing localized IMP delivery for clinical translation.

## Materials and Methods

### Antibodies, Reagents, and Resources

The antibodies, reagents, and resources used in this study are provided in Table 1.

**Table 1:** Key Antibodies, Reagents, and Resources

Reagent or resource	Source	Identifier
Antibodies		
Rabbit Anti-AIM2 polyclonal antibody	Absin	Cat.# abs125828
Phospho-Smad2/3 rabbit polyclonal antibody	Absin	Cat.# abs130992
Rabbit anti-TNFα polyclonal antibody	Absin	Cat.# abs131997
Anti-GSDMD antibody	Abcam	Cat.# ab209845
Rabbit anti-mouse IgG	Abcam	Cat.# ab6728
CD11b rabbit mAb	Abcam	Cat.# ab8878
GAPDH rabbit mAb	Abclonal	Cat.# a19056
Cleaved caspase-1 rabbit mAb	Cell Signaling Technology	Cat.# 89332S
IL-1β rabbit mAb	Cell Signaling Technology	Cat.# 31202
Smooth muscle actin polyclonal antibody	Proteintech	Cat.# 14395
Collagen type I polyclonal antibody	Proteintech	Cat.# 14695
FITC conjugated donkey anti-rabbit IgG (H+L)	Servicebio	Cat.# GB22403
Cy3 conjugated goat anti-rabbit IgG (H+L)	Servicebio	Cat.# GB21303
Anti-collagen I rabbit polyclonal antibody	Servicebio	Cat.#GB11022
Anti-alpha smooth muscle actin rabbit polyclonal antibody	Servicebio	Cat.#GB111364
Chemicals and recombinant proteins		
RNase-free DTT	Ansiang	Cat.# 5-750
Formalin	Ansiang	Cat.# 5-171
Fetal bovine serum	AusGenX	Cat.# No.FBS500-S
PVDF membranes	EMD Millipore	Cat.#: C3117
RIPA buffer	FUDE	Cat.# FD008
HRP substrate	EMD Millipore	Cat.#: 18-160
2,4,6-trinitrobenzenesulfonic acid (TNBS)	Sigma	Cat.# P2297
Maxpar fix and perm buffer	Fluidigm	Cat.# S00092
HE dye solution	Servicebio	Cat.# G1005
Sirius Red staining solution	Servicebio	Cat.#G1018
Hematoxylin staining solution	Servicebio	Cat.# G1004
Eagle’s medium (DMEM)	GIBCO	Cat.# 30030
DMEM	GLPBIO	Cat.# 11320033
Collagenase	GLPBIO	Cat.# 17104019
transfection reagent RNAi-Mate	GenePharma	Cat.# G04001
Trypsin	HARVEYBIO	Cat.# 9002-07-7
Physiological saline	NucleoTech	Cat.# NS23032-10
Protein-free rapid blocking buffer	EpiZyme	Cat.# PS108P
Ethanol anhydrous	Merda	Cat.# M155707-25L
Isoflurane	RWD	Cat.# R510-22
Bovine serum albumin	Servicebio	Cat.#GC305006
Citric acid antigen-retrieval solution	Servicebio	Cat.#G1202
Ethylenediaminetetraacetic acid disodium salt dihydrate	Sigma	Cat.#: E5134-500G
DNase I	Sigma-Aldrich	Cat.# AMPD1
Paraformaldehyde	Sigma-Aldrich	Cat.# P6148
Phosphate buffered saline	Solarbio	Cat.# P1010
Propidium iodide	Solarbio	Cat.# P8080
Gentle cell dissociation reagent	Stemcell Technologies	Cat.# 7174
EasySep Releasable RapidSpheres	Stemcell Technologies	Cat.# 50201
Imperatorin	Macklin	Cat. # No. I875280
Penicilin-streptomycin liquid	Solarbio	Cat.# P1400
TSV405 TSnanofect V2	TSINGKE	Cat. # No. TSV405
Protein G immunoprecipitation magnetic beads	Selleck	Cat. # B23201
SDS-PAGE loading buffer	YEASEN	Cat. # 20315ES05
Poly(deoxyadenylic-thymidylic) acid sodium salt (poly(dA:dT))	Sigma	Cat. # P0883
Lipopolysaccharide	Macklin	Cat. # L861706
CELLSAVING	NCM Biotech	Cat. # C40100
RNase-free water	Takara	Cat.# No.9012
Xylene	TCI	Cat.# D0429
DAPI	Thermo Fisher Scientific	Cat.# 62248
Agarose	Yeasen	Cat.# 10208ES60
5× HieffCanace PCR Master Mix	Yeasen	Cat.# 10137ES08
Phosphatase inhibitor cocktail	Yeasen	Cat.# 20109ES20
Experimental models: cell lines		
J774A.1	FUHENG	Cat.# FH0329
Experimental models: organisms/strains		
C57BL/6 mice	This paper	N/A
C57BL/7 mice (Gsdmd^-/-^)	This paper	N/A
E.Z.N.A. soil DNA Kit	Omega Bio-tek	Cat.# M1768
BCA kit	Beyotime	Cat.# P0009
RNAiso Plus	Takara	Cat.# No. 9108
IL-1β ELISA kit	EIAAB	Cat.# E0563m
TGF-β ELISA kit	EIAAB	Cat.# E0124h
RT-PCR kit	Takara	Cat.# RR014
PAGE Gel quick preparation kit	Yeasen	Cat.# 20324ES62
Mouse tissue lysis component	Yeasen	Cat.# 19697ES50
Hifair Ⅲ reverse transcriptase	Yeasen	Cat.# 14601ES10
Deposited data		
RNA-seq (J744A.1, Ctrl and IMP)	This manuscript	PRJCA020332
Software and algorithms		
ImageJ	NIH	https://imagej.nih.gov/ij/
Prism software v9.0	GraphPad Software	https://www.graphpad.com/

### Human Samples

Mucosal tissues of ileocecal valves were collected from patients with CD and HCs attending the Department of Gastroenterology of Nanfang Hospital. Disease diagnoses were assessed by a standard combination of clinical, endoscopic, and histological criteria. Mucosal tissue samples were collected from consenting individuals during routine endoscopy according to the protocols approved by the Ethics Committee of Nanfang Hospital of Southern Medical University. Demographic characteristics are shown in Table 2.

**Table 2:** Demographic Characteristics of the Study Population

	CD	Controls
No.	15	15
Male, n (%)	7 (46.6)	8 (53.3)
Age, *years*	26.25 ± 8.75 (14–56)	28.75 ± 9.25 (20–58)
Disease duration, *months*	30.6 ± 12.8 (2–60)	_
Age at diagnosis, *years*	20.00 ± 6.5 (14–32)	_

NOTE. Data are presented as number (%) or mean ± standard deviation (range).

CD, Crohn’s disease.

### Mouse Models

WT and *Gsdmd*^-/-^mice (C57BL/6 strain) were bred and maintained in a pathogen-free facility, receiving standard chow and water ad libitum. The *Gsdmd*^-/-^ mice were kindly provided by Professor Feng Shao (National Institute of Biological Sciences, Beijing, China). The *Gsdmd*^-/-^ mice were conducted using cohoused WT littermates as controls. All animal-related experimental protocols were approval by the Institutional Animal Care and Use Committee of Southern Medical University (Approval NO. L2018231).

Our study examined male mice because male animals exhibited less variability in phenotype. A TNBS-induced colitis and fibrosis model was established according to the published procedures.^30^ Briefly, 8- to 10-week-old male *Gsdmd*^-/-^ mice and their WT littermates were pretreated with 100 μL TNBS presensitization solution (1% TNBS dissolved in 4:1acetone: olive oil solution), which was applied to shaved back skin on day −7. These mice were lightly anesthetized with isoflurane. Next, they were intrarectally injected with 100 μL 2.5% TNBS (controls were injected with 100 μL PBS) through a flexible catheter inserted 3.5 cm into the rectum on days 0, 7, 14, 21, 28, and 35. In the IMP-treated groups, WT mice were intrarectally injected with 100 μL 3 mM IMP 1 day before TNBS enema. During experiments, body weight was monitored daily. Colon tissues were embedded in paraffin and stained with hematoxylin and eosin (H&E), and histologic scores were assessed as in the published procedures.^30^ Sirius red staining was performed and calculated by ImageJ software to quantify the fibrosis degree of colon tissues.

### Cell Culture and Transfection

J774A.1 cells were from Fuheng Biology Corporation and were cultured in Dulbecco’s modified Eagle’s medium (DMEM, Gibco) supplemented with 10% fetal bovine serum (FBS) in 5% CO_2_ at 37°C. These cells were transfected with 1 μg/μL LPS plus 2.5 ug/mL poly (dA: dT) for 8 hours to induce pyroptosis.

### Western Blotting

Western blotting procedures were performed as previously described.^31^ The density of protein bands was analyzed and quantified by ImageJ software. The specific antibodies used in this study are provided in Table 1.

### IHC Analysis

IHC analysis was conducted to evaluate the expression levels of AIM2 in paraffin-embedded tissues using specific anti-AIM2 antibodies. Protein expression levels were analyzed and scored by professional pathologists with Image-Pro Plus software.

### Immunofluorescence Staining

The tissue sections were fixed in 4% neutral buffered formalin for 5 minutes and subjected to immunofluorescence staining using primary antibodies targeting CD11b, F480, GSDMD-NT, IL-1β, AIM2, Collagen Ⅰ, and α-SMA. Subsequently, the sections were incubated with a biotinylated secondary antibody against immunoglobulin (Ig) and streptavidin-fluorescein isothiocyanate (FITC). Nuclei were counterstained with 4',6-diamidino-2-phenylindole (DAPI). Ten randomly selected fields of view were captured from each slide using a fluorescent microscope. The fluorescence intensities of each field were quantified using ImageJ software. The specific antibodies used for immunofluorescence staining are listed in Table 1.

### RNA Sequencing

RNA sequencing (RNA-seq) was employed to evaluate the mRNA expression levels in J744A.1 cells, with and without IMP treatment. For RNA extraction, cells were lysed in 1000 μL of Buffer RLT (with the addition of 10 μL β-ME per 1 mL of Buffer RLT prior to use) and thoroughly mixed by vortexing or pipetting. The selection of the sample quality control protocol was based on sample types and specific requirements. Reverse transcription was conducted to generate first-strand cDNA using SMART technology. Polymerase chain reaction (PCR) amplification was subsequently performed to preamplify the cDNAs. Qualified amplified cDNAs were then utilized for library construction using a transposon-based method.

### LDH Release Assay

J774A.1 cells were seeded in 6-well plates and were stimulated with 50 μM IMP or control medium for 6 hours, and then transfected with LPS (1 μg/μL) plus poly (dA: dT) (2.5 ug/mL) for 8 hours to induce pyroptosis. The culture supernatants were collected, and the levels of LDH in the supernatants were quantified using an LDH assay kit (Solarbio).

### PI Staining

PI staining technique was used to observe the morphological pattern of IMP improving pyroptosis. Properly treated J774A.1 cells were seeded into 6-well plates and then stained with 100 μL PI (P8080-10mg, Solarbio) at room temperature for 15 minutes in the dark and then washed twice with PBS. The results were visualized under a fluorescence microscope (BX63; Olympus) at 20× magnification. The excitation and emission wavelengths were 535 nm and 614 nm.

## Enzyme-linked Immunosorbent Assay

J774A.1 macrophages were treated with or without 50 μM IMP for 6 hours following transfection with 1 μg/μL LPS plus 2.5 ug/mL poly (dA: dT) for 8 hours, and then their culture supernatants were collected for IL-1β assay. In addition, their culture supernatants were collected to stimulate the human colon fibroblast cell line CCD-18Co. After 24 hours, CCD-18Co cells were changed to a new medium and cultured for 12 hours, and subsequently, their culture supernatants were collected for TGF-β assay.

### Statistical Analysis

Statistical analysis was performed using GraphPad Prism 9 software. All variables are expressed as means ± standard error of the mean (SEM) or means ± standard deviation (SD), as noted in the figure legends. One-way analysis of variance (ANOVA) test was used for comparisons among 3 or more groups, and unpaired Student’s *t*-test for comparison between 2 groups. Statistical significance was defined as *∗P <* .05, *∗∗P <* .01, *∗∗∗P <* .001, and *∗∗∗∗P <* .0001.
